# A divergent role for estrogen receptor-beta in node-positive and node-negative breast cancer classified according to molecular subtypes: an observational prospective study

**DOI:** 10.1186/bcr2139

**Published:** 2008-09-04

**Authors:** Flavia Novelli, Michele Milella, Elisa Melucci, Anna Di Benedetto, Isabella Sperduti, Raffaele Perrone-Donnorso, Letizia Perracchio, Irene Venturo, Cecilia Nisticò, Alessandra Fabi, Simonetta Buglioni, Pier Giorgio Natali, Marcella Mottolese

**Affiliations:** 1 Pathology Department, Regina Elena Cancer Institute, Via Elio Chianesi 53, 00144 Rome, Italy; 2 Medical Oncology Department, Regina Elena Cancer Institute, Via Elio Chianesi 53, 00144 Rome, Italy; 3 Regina Elena Cancer Institute, Via delle Messi d'oro 156, 00158 Rome, Italy

## Abstract

**Introduction:**

Estrogen receptor-alpha (ER-α) and progesterone receptor (PgR) are consolidated predictors of response to hormonal therapy (HT). In contrast, little information regarding the role of estrogen receptor-beta (ER-β) in various breast cancer risk groups treated with different therapeutic regimens is available. In particular, there are no data concerning ER-β distribution within the novel molecular breast cancer subtypes luminal A (LA) and luminal B (LB), HER2 (HS), and triple-negative (TN).

**Methods:**

We conducted an observational prospective study using immunohistochemistry to evaluate ER-β expression in 936 breast carcinomas. Associations with conventional biopathological factors and with molecular subtypes were analyzed by multiple correspondence analysis (MCA), while univariate and multivariate Cox regression analysis and classification and regression tree analysis were applied to determine the impact of ER-β on disease-free survival in the 728 patients with complete follow-up data.

**Results:**

ER-β evenly distributes (55.5%) across the four molecular breast cancer subtypes, confirming the lack of correlation between ER-β and classical prognosticators. However, the relationships among the biopathological factors, analyzed by MCA, showed that ER-β positivity is located in the quadrant containing more aggressive phenotypes such as HER2 and TN or ER-α/PgR/Bcl2^- ^tumors. Kaplan-Meier curves and Cox regression analysis identified ER-β as a significant discriminating factor for disease-free survival both in the node-negative LA (*P *= 0.02) subgroup, where it is predictive of response to HT, and in the node-positive LB (*P *= 0.04) group, where, in association with PgR negativity, it conveys a higher risk of relapse.

**Conclusion:**

Our data indicated that, in contrast to node-negative patients, in node-positive breast cancer patients, ER-β positivity appears to be a biomarker related to a more aggressive clinical course. In this context, further investigations are necessary to better assess the role of the different ER-β isophorms.

## Introduction

It has been shown that longer exposure to estrogen results in an increased risk of developing breast cancer (BC) and endogenous estrogens are thought to play a major role in BC carcinogenesis [1]. Moreover, in BC, estrogen receptor-alpha (ER-α) and progesterone receptor (PgR) are well-established biomarkers capable of predicting the likelihood of relapse/progression in response to endocrine therapy. The identification of a second type of ER, named estrogen receptor-beta (ER-β) [2], has prompted the re-evaluation of the model of estrogen action. To this end, a number of studies have been conducted retrospectively on selected series of invasive BC to evaluate the predictive value of ER-β in patients submitted to endocrine therapy [3]. Unlike ER-α, antiestrogen-occupied ER-β can activate transcription via nongenomic ER signaling pathways that involve the activation of cytoplasmic signal transduction cascades such as the Src/ERK and the PI3K/Akt pathways [4]. ER-α and ER-β can mediate the biological effects of estrogens and antiestrogens by modulating the expression of specific target genes. At present, however, limited information is available concerning the differential modulation of gene expression from either ER-α or ER-β, which share a high degree of homology in the DNA-binding domain but differ considerably in the NH_2_-terminal region and, to a lesser extent, in the ligand-binding domain. Because of this lack of sequence similarity, it has been suggested that the two receptors might perform distinct functions [5].

Another important steroid receptor involved in BC progression is the PgR, which plays a pivotal role in the action of progestins in target cells and tissues. In invasive BC, PgR expression is generally regarded as a marker of an intact ER-α signaling pathway [6]. ER-α and PgR positivities correlate with favorable prognostic features and are predictors of response to hormonal therapy (HT), both in the adjuvant setting and in advanced disease. In contrast, much less is known regarding the contribution of ER-β to estrogen-driven responses [7] or its prognostic/predictive role in different early BC risk groups treated with different chemotherapeutic/hormonal regimens. This picture has recently been complicated further by the introduction of gene profiling approaches [8] and by the widespread application of a novel BC classification based on the immunohistochemistry (IHC) phenotypic patterns identified by a few protein biomarkers, namely ER-α, PgR, HER2, epidermal growth factor receptor (EGFR), and low-molecular-weight cytokeratins [9]. According to the expression of such markers, BC can now be divided into four main subtypes that have distinct behavior in terms of prognosis and response to therapy [8]: luminal A (LA) and luminal B (LB), characterized by high expression of ER-α; triple-negative (TN), characterized by EGFR and/or by some basal epithelial markers such as cytokeratin 5 positivity; and HER2, characterized by the lack of hormonal receptors. To date, there are no published data concerning the distribution of ER-β among these different molecular subtypes of BC. The aims of the present study were (a) to prospectively evaluate the relationship between ER-β and a number of established biopathological parameters in an observational prospective series of 936 BC patients and (b) to analyze the impact of ER-β expression on clinical outcome and on the response to different therapeutic regimens, taking into account the novel molecular classification.

## Materials and methods

### Patient characteristics

ER-β was analyzed by IHC in a series of 936 BC patients subjected to breast surgery at the Regina Elena Cancer Institute (Rome, Italy) between 2001 and 2005. ER-β expression was routinely determined at the time of surgical treatment along with other conventional biological factors before any adjuvant therapy was planned. Patients were subjected to modified radical mastectomy or breast-conserving surgery (quadrantectomy). Radiotherapy was offered to all patients treated with quadrantectomy and to patients with lymph node metastases treated with modified radical mastectomy. Follow-up data were obtained from hospital charts and by corresponding with the referring physicians. The clinicopathological characteristics of these patients are summarized in Table 1. Seven hundred sixty-seven of 936 patients with invasive BC with a median follow-up of 50 months (range 1 to 108 months) were analyzed for disease-free survival (DFS). The remaining 169 patients (18%) were excluded from DFS analysis since follow-up for disease recurrence was not available or they were treated with chemotherapy (CHT) before surgery. The subgroup analyzed for DFS included 665 (86.7%) invasive ductal carcinomas, 9 tubular carcinomas (1.2%), 87 invasive lobular carcinomas (11.3%), and 6 medullary carcinomas (0.8%). In this series, 58.1% were ER-β^+^, 69.4% ER-α^+^, 60.6% PgR^+^, and 31.9% HER2^+ ^(19.9% score 2+ and 12% score 3+) (data not shown). We also studied ER-β distribution in the four different molecular subtypes: LA (ER-α/PgR^+ ^and HER2^-^, n = 447), LB (ER-α/PgR^+ ^and HER2^+^, n = 166), TN (ER-α/PgR^- ^and HER2^-^, n = 159), and HS (ER-α/PgR^- ^and HER2^+^, n = 164). Tumors were graded according to Bloom and Richardson and staged according to the Unione Internationale Contre le Cancer tumor-node-metastasis system criteria and histologically classified according to the World Health Organization [10]. The study was reviewed and approved by the ethics committee of the Regina Elena National Cancer Institute, and written informed consent was obtained from all patients.

**Table 1:** Clinicopathological characteristics of 936 invasive breast carcinomas

Characteristic	Number of cases	Percentage
Menopausal status		
Premenopause	668	71.4
Postmenopause	268	28.6
Histotype		
Tubular carcinoma	14	1.5
Invasive ductal carcinoma	825	88.2
Invasive lobular carcinoma	91	9.7
Other	6	0.6
Histologic grade		
Grade 1	151	16.0
Grade 2	493	53.0
Grade 3	292	31.0
Lymph node status		
Negative	539	58.0
Positive	397	42.0
Tumor size		
T1	579	62.0
T2	290	31.0
T3,4	67	7.0
Estrogen receptor-beta		
Negative	416	44.0
Positive	520	56.0
Estrogen receptor-alpha		
Negative	278	30.0
Positive	658	70.0
Progesterone receptor		
Negative	368	39.0
Positive	568	61.0
HER2		
Negative (0/1+)	635	68.0
Positive (2+/3+)	183	32.0
Ki67 expression		
Low	529	56.0
High	407	44.0
p53		
Negative	714	76.0
Positive	222	23.0
Bcl2		
Negative	297	32.0
Positive	639	68.0

### Immunohistochemistry

Formalin-fixed paraffin-embedded breast specimens were cut on SuperFrost Plus slides (Menzel-Gläser, Braunschweig, Germany). Antigen retrieval was performed by microwave at 430 W (1 mM citrate buffer, pH 6.0) for two cycles of 10 minutes each and one of 5 minutes for anti-ER-β monoclonal antibodies (MoAbs) and by thermostatic bath at 96°C (10 mM/L citrate buffer, pH 6) for 40 minutes for ER-α, PgR, HER2, p53, Bcl2, and Ki67. Sections were incubated with the anti-ER-β MoAbs PPG5/10 (ER-β1, dilution 1:15; GeneTex, Inc., Prodotti Gianni, Milan, Italy) and 14C8 (ER-β total, dilution 1:25; AbCam, Valter Occhiena s.r.l., Turin, Italy) overnight at 4°C, with the anti-ER-α MoAb 6F11 (Novocastra, Menarini, Florence, Italy), the anti-PgR MoAb 1A6 (Menarini), the anti-Ki67 MoAb MIB-1 (Dako, Milan, Italy), the anti-p53 MoAb DO7 (Dako), the anti-Bcl2 MoAb 124 (Dako), and the anti-HER2 polyclonal antibody (A0485; Dako) for 30 minutes at room temperature. Positive and negative controls were included for each antibody and in each batch of staining. The immunoreactions were revealed by a streptavidin-biotin-enhanced peroxidase system (Super Sensitive Link-Label IHC Detection System; BioGenex, Space, Milan, Italy) using 3-amino-9-ethylcarbazole (Dako) as chromogenic substrate. ER-β was defined as negative (ER-β^-^) when a weak staining reaction was observed in less than 20% of carcinoma cell nuclei and as positive (ER-β^+^) when a moderate/strong staining reaction was observed in 20% to 100% of neoplastic cell nuclei. This cutoff, which was in agreement with Shaaban and colleagues [11] and Gruvberger-Saal and colleagues [3], was generated using the classification and regression tree (C&RT) analysis (see Statistical methods). We introduced three variables (nodal status, ER-β expression, and relapses) into the model. The model indicated that, in node-negative patients, the highest percentage of relapses occurred when ER-β was positive in less than 17.5% of neoplastic cells whereas, in node-positive patients, the highest percentage of relapses occurred when ER-β was positive in more than 12.5% of neoplastic cells (Additional file 1). ER-α, PgR, and p53 were considered positive when greater than 10% of the neoplastic cells showed a distinct nuclear immunoreactivity whereas Ki67, based on the median value of our series, was regarded as high if greater than 15% of the cell nuclei were immunostained. Bcl2 was recorded as positive when tumor cells exhibited a strong homogeneous cytoplasmic immunoreaction in more than 30% of neoplastic cells [12]. HER2 overexpression was determined as defined in the guide of the HercepTest kit (Dako): scores of 0 or 1+ were considered negative, 2+ weak positive, and 3+ strong positive. Evaluation of the immunohistochemical results, blinded to all patient data, was performed independently and in blinded manner by two investigators (M Mottolese and FN).

### Statistical methods

Correlation among MoAbs anti-ER-β, PPG5/10, and 14C8 was estimated using the kappa test, whereas the correlation between ER-β and the biopathological characteristic variables was tested by the Pearson chi-square test. Multiple correspondence analysis (MCA), a descriptive/exploratory technique designed to analyze simple two-way and multi-way tables, was used to identify prognostic biological profiles. The results provide information that is similar in nature to that produced by factor analysis techniques and make it possible to explore the structure of categorical variables included in the table. The most common kind of this type is the two-way frequency cross-tabulation table [13,14]. This representation aims to visualize the similarities and/or differences of profiles, simultaneously identifying those dimensions that contain the majority of the data variability. The positions of the points in the MCA graph are informative. Categories plotting close to each other are statistically related and are similar with regard to the pattern of relative frequencies and this association is statistically valuable (Lebart's statistic) when the points are located far from the origin of the graph which represents a mean uninformative profile. ER-β, p53, Ki67, Bcl2, hormonal receptors, or the BC subtypes were the variables of major interest for the purpose of our study. These factors were introduced in each analysis as active variables whereas the pathological factors (tumor size [T], lymph node status [N], and histological grade [G]) were introduced as supplementary variables. MCA provides a graphical representation of the active and supplementary variables projected on a plane formed by axes 1 and 2, which accounted for 67.6% of total variability, reproducing quite a significant percentage of the total chi-square value of the multi-way frequency table. C&RT analysis, a type of decision tree methodology, is a nonparametric statistical procedure that identifies mutually exclusive and exhaustive subgroups of a population whose members share common characteristics that influence the dependent variable of interest. C&RT uses a binary recursive partitioning method that produces a decision tree that identifies subgroups of patients with a higher likelihood of being found positive in a test for a disease state. For analytical purposes, the patients are split into two groups: a CHT-treated group that included anthracycline (AC) and no AC regimens and an HT-treated group. The procedure examines all possible independent or splitting variables and selects the one that produces the most different binary groups compared with the dependent variable according to a predetermined splitting criterion. The parent node, containing the entire sample, branches into two child nodes according to the independent variable. Within each of the two child nodes, the tree-growing methodology continues by assessing each of the remaining independent variables to determine which variable results in the best split according to the chosen criterion. The improvement in prediction was evaluated by the Gini coefficient. At the point where no further split is made, a terminal node is created [15]. For the purpose of our study, DFS was considered as a measure of outcome. DFS was calculated from the date of tumor diagnosis to the date of first recurrence, including contralateral carcinomas, local relapses, or distant metastases (Additional file 2). Patients without recurrence were censored at the time of last follow-up. The DFS curves were estimated by the Kaplan-Meier product-limit method. The log-rank test was used to assess differences between subgroups, and significance was defined as a *P *value of less than 0.05. A multivariate Cox proportional hazard model was also developed using stepwise regression (forward selection) with predictive variables that were significant in the univariate analyses. The enter limit and remove limit were *P *= 0.10 and *P *= 0.15, respectively. The SPSS (version 14.0) statistical program (SPSS Inc., Chicago, IL, USA) was used for analyses.

## Results

### Relationship among estrogen receptor-beta and biopathological parameters

**Figure 1:**
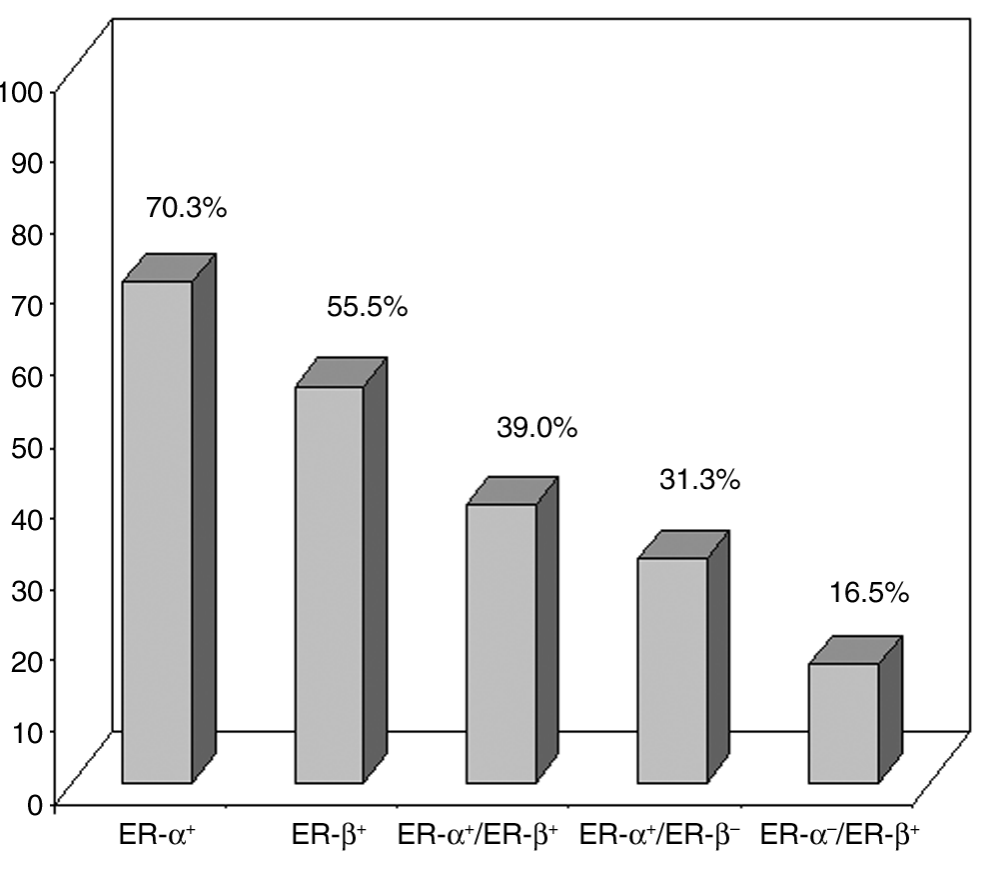
**ER-alpha (ER-α) and ER-beta (ER-β) distribution in breast carcinomas**. Estrogen receptor-alpha (ER-α) and estrogen receptor-beta (ER-β) frequency and coexpression in 936 invasive breast carcinomas in an observational prospective study.

**Figure 2:**
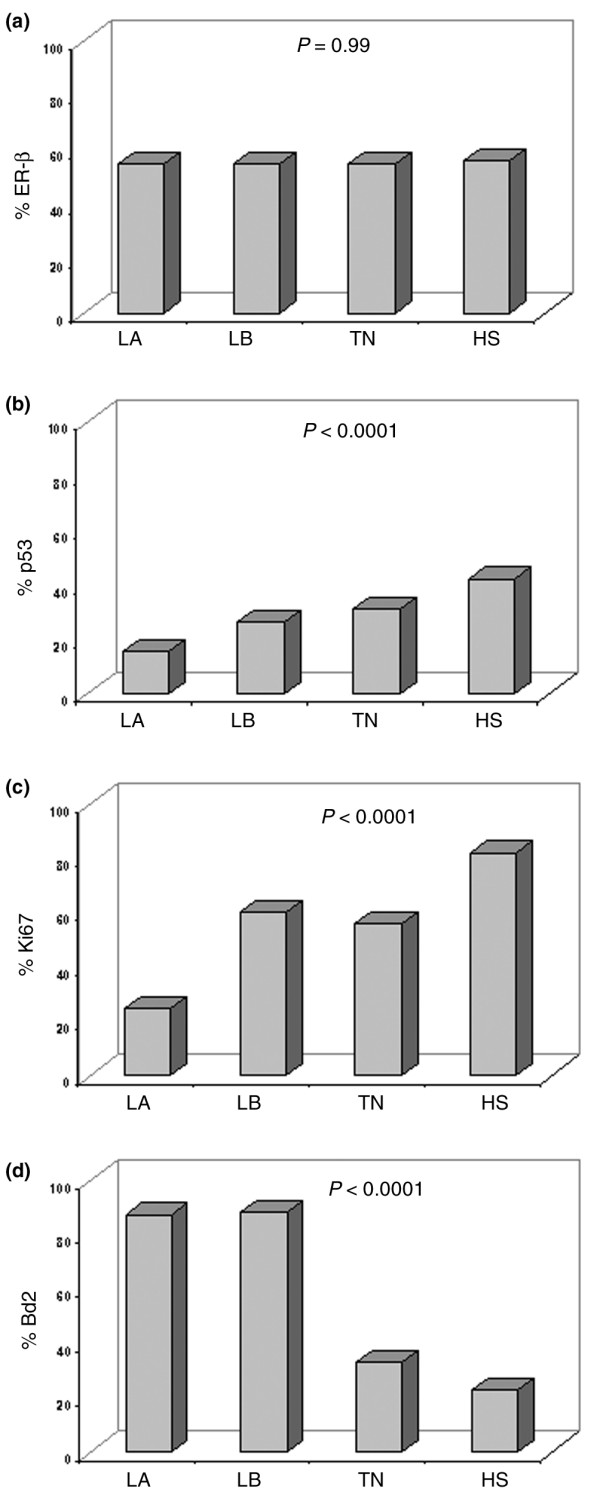
**ER-beta (ER-β), p53, Ki-67 and Bcl2 distribution within BC molecular subtypes**. Estrogen receptor-beta (ER-β) evenly distributes (55% to 56%, *P *= 0.99) across the four molecular subtypes **(a)**, whereas the percentages of p53 **(b) **and Ki67^+ ^**(c) **tumors significantly increased (*P *< 0.0001) and the percentage of Bcl2 significantly decreased (*P *< 0.0001) moving from the luminal A (LA) phenotype to luminal B (LB), triple-negative (TN), and HER2 (HS) **(d)**.

In our series of 936 BC patients, 56% of the tumors stained positive for ER-β1 as detected by MoAb PPG5/10. These data were also confirmed using the 14C8 MoAb-directed anti-total ER-β (data not shown) [16], which, in our series, showed a very good correlation with PPG5/10 (κ = 0.80, 95% CI 69 to 92, *P *< 0.0001). Results reported hereafter refer to those obtained with the PPG5/10 MoAb. No significant correlation was observed between ER-β expression and the other parameters analyzed. In contrast, ER-α and PgR were directly related to each other (*P *< 0.0001) and to Bcl2 (*P *< 0.0001) and inversely correlated with T, G, p53, HER2, and Ki67 (*P *< 0.0001), as expected from previous reports [11,17]. ER-α, but not PgR, expression was also significantly associated (*P *= 0.04) with negative nodal status. The same analysis, performed in the cohort of 767 patients with known follow-up, gave superimposable results (data not shown). As shown in Figure 1, 70.3% of cases were ER-α^+ ^and 55.5% were ER-β^+^. ER-α and ER-β were coexpressed in 39.0% of cases whereas 31.3% of breast carcinomas were ER-α^+ ^and ER-β^- ^and 16.5% were ER-α^- ^and ER-β^+^. When ER-β expression was analyzed according to the novel BC classification (Figure 2a), we found that the receptor evenly distributes (*P *= 0.99) among the four molecular subtypes: indeed, ER-β stained positive in 54.7% of LA, 55.4% of LB, 55.3% of TN, and 56.3% of HS. In contrast, the percentage of p53 and Ki67^+ ^tumors significantly increased (*P *< 0.0001) (Figure 2b,c) and the percentage of Bcl2^+ ^tumors significantly decreased moving from the LA phenotype to LB, TN, and HS (*P *< 0.0001) (Figure 2d).

### Multiple correspondence analysis

**Figure 3:**
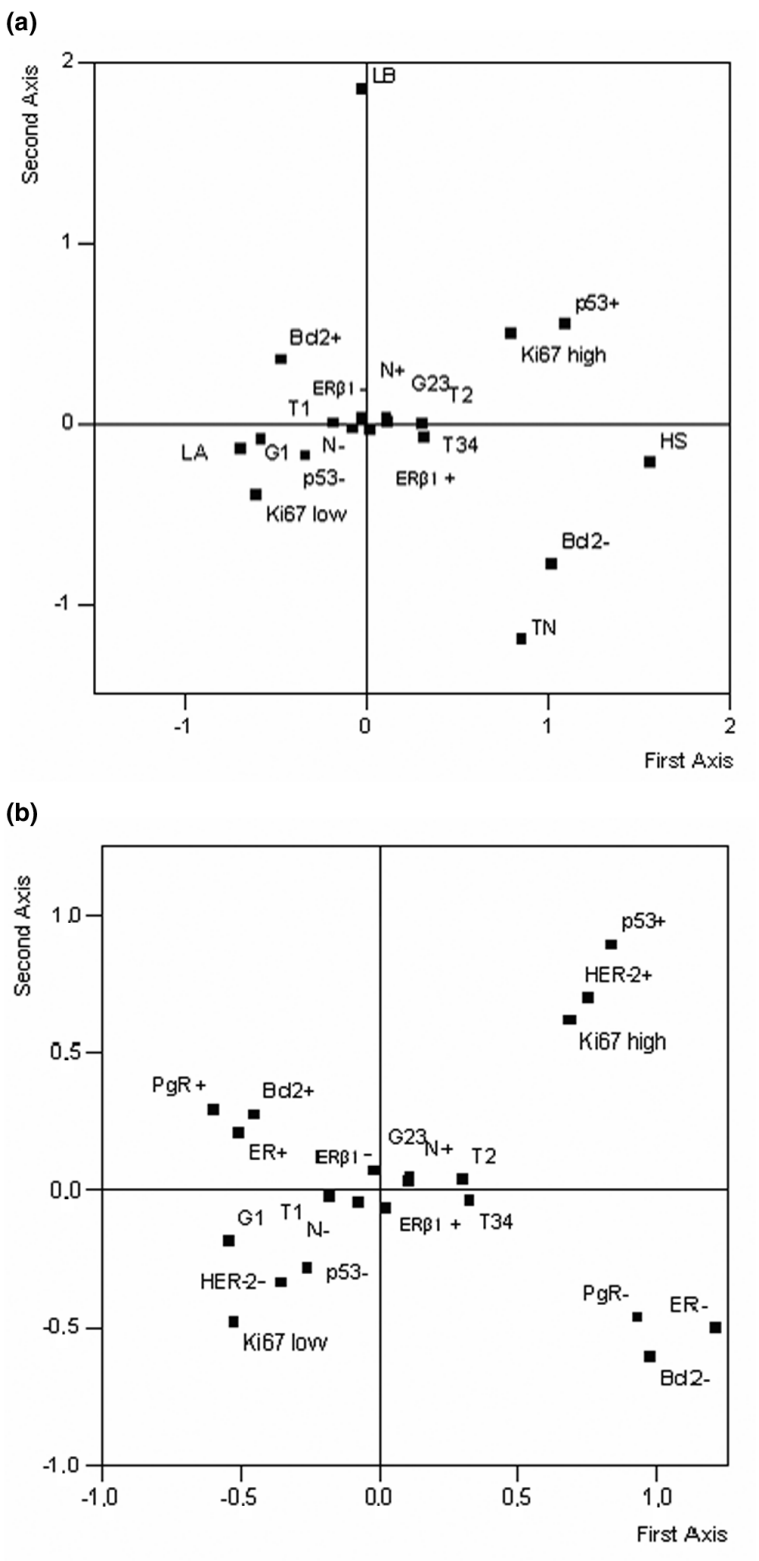
**Multiple correspondence analysis of the 936 breast carcinomas**. Multiple correspondence analysis of the 936 invasive breast carcinomas. Estrogen receptor-beta (ER-β) positivity is located in the quadrant containing more aggressive phenotypes such as HER2 (HS) and triple-negative (TN) **(a) **or estrogen receptor-alpha, progesterone receptor (PgR), and Bcl2-negative tumors **(b)**. LA, luminal A; LB, luminal B; N-, node-negative; N^+^, node-positive.

As shown in Figure 3, the complex interrelationships among the biopathological variables considered in our study, either clustered into phenotypic subtypes (Figure 3a) or considered individually (Figure 3b), can best be evaluated by using MCA. Using this analysis, it is possible to visualize the association of biological factors (ER-α, PgR, HER2, Ki67, p53, and Bcl2) with ER-β and study their link with conventional pathological factors (N, G, and T). As shown in Figure 3a, along the first axis, the test demonstrates the contrast between high Ki67/p53^+ ^tumors (upper right quadrant) and LA low Ki67/p53^- ^tumors (lower left quadrant), suggesting that these two groups represent biopathologically and statistically distinct entities as their defining parameters appear close to each other directly, far from the origin, and diagonally opposite. Similarly, the second axis clearly differentiates Bcl2^+ ^tumors/LB subtype (upper left quadrant) from Bcl2^- ^tumors/HER2^+ ^and TN (lower right quadrant), indicating that these two groups, far from the origin and diagonally opposite, can be statistically correlated also. ER-β^+ ^(55.5%), though close to the origin, is located in the same quadrant (lower right quadrant) as Bcl2^-^/HER2 subtype/LB subtype tumors, whereas ER-β^- ^(44.5%) is located in the opposite upper left quadrant. Figure 3b shows the contrast between ER-α/PgR/Bcl2^- ^tumors (lower right quadrant) and ER-α/PgR/Bcl2^+ ^tumors (upper left quadrant), whereas the second axis clearly differentiates p53^+^/HER2^+^/high Ki67 tumors (upper right quadrant) from p53^-^/HER2^-^/low Ki67 tumors (lower left quadrant). Consistent with the lack of correlation with other variables and with its even distribution across different molecular subtypes, ER-β^+ ^(55.2%) or ER-β^- ^(44.8%) did not discernibly cosegregate with any of the other biopathological factors considered.

### Impact of estrogen receptor-beta expression on disease outcome

#### Classification and regression tree analysis

**Figure 4:**
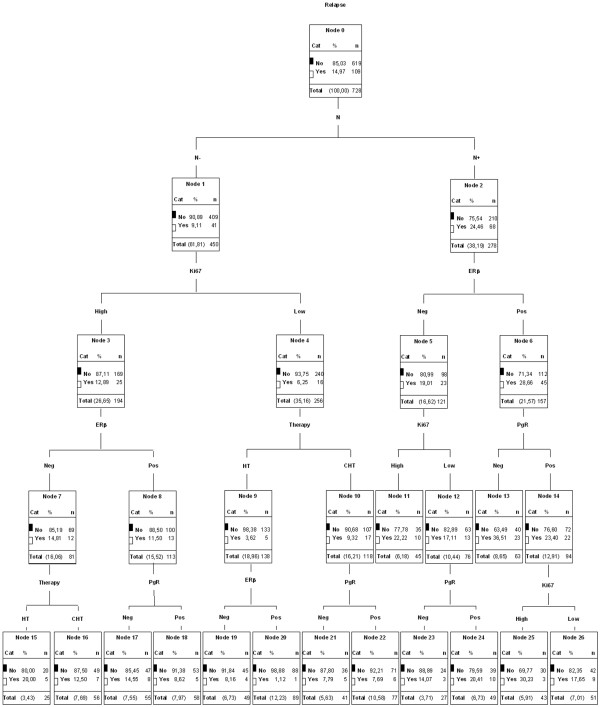
**Classification and regression tree analysis (C&RT) of the 728 patients with known follow-up**. Classification and regression tree analysis of 728 patients with known follow-up predicts which patient belongs to which specific class (good or poor clinical outcome) on the basis of clinical and biopathological information. Diagram shows diagnostic algorithm generated by AnswerTree 3.1 software, Statistical Package for the Social Sciences (SPSS Inc., Chicago, IL, USA). CHT, chemotherapy; ER-β, estrogen receptor-beta; HT, hormonal therapy, N-, node-negative; N^+^, node-positive; No, absence of recurrences; PgR, progesterone receptor; Yes, presence of recurrences.

The prognostic impact of ER-β and other biopathological/clinical parameters on DFS was further assessed in a cohort of 767 patients with complete follow-up data. Thirty-nine patients who received no systemic adjuvant treatment were excluded from further analysis. Among the remaining 728 patients, 231 (32%) received exclusive HT, 249 (34%) AC-based CHT, and 248 (34%) non-AC-based CHT. At a median follow-up of 50 months (range 1 to 108 months), a total of 109 out of 728 patients (15%) showed progressive disease (Additional file 2). C&RT analysis for factors influencing DFS was performed including all of the biopathological (ER-β, ER-α, PgR, p53, Ki67, Bcl2, T, N, and G) and clinical (age, menopausal status, therapy, and DFS) parameters into the model. As graphically described in Figure 4, the parent node contains the entire sample of 728 patients. The first splitting node was nodal status (N). Within the node-negative category (on the left of the graph), Ki67 was the next splitting node, followed by ER-β status and type of therapy received in the high and low Ki67 categories, respectively; in the HT node, ER-β expression was associated with a lower number of relapses and better DFS. Conversely, the node-positive category that splits according to ER-β expression showed a higher number of relapses among ER-β^+ ^patients: ER-β^+ ^cases further split by PgR expression, with lack of PgR correlating with a worse outcome in terms of DFS.

#### Univariate and multivariate analysis

**Figure 5:**
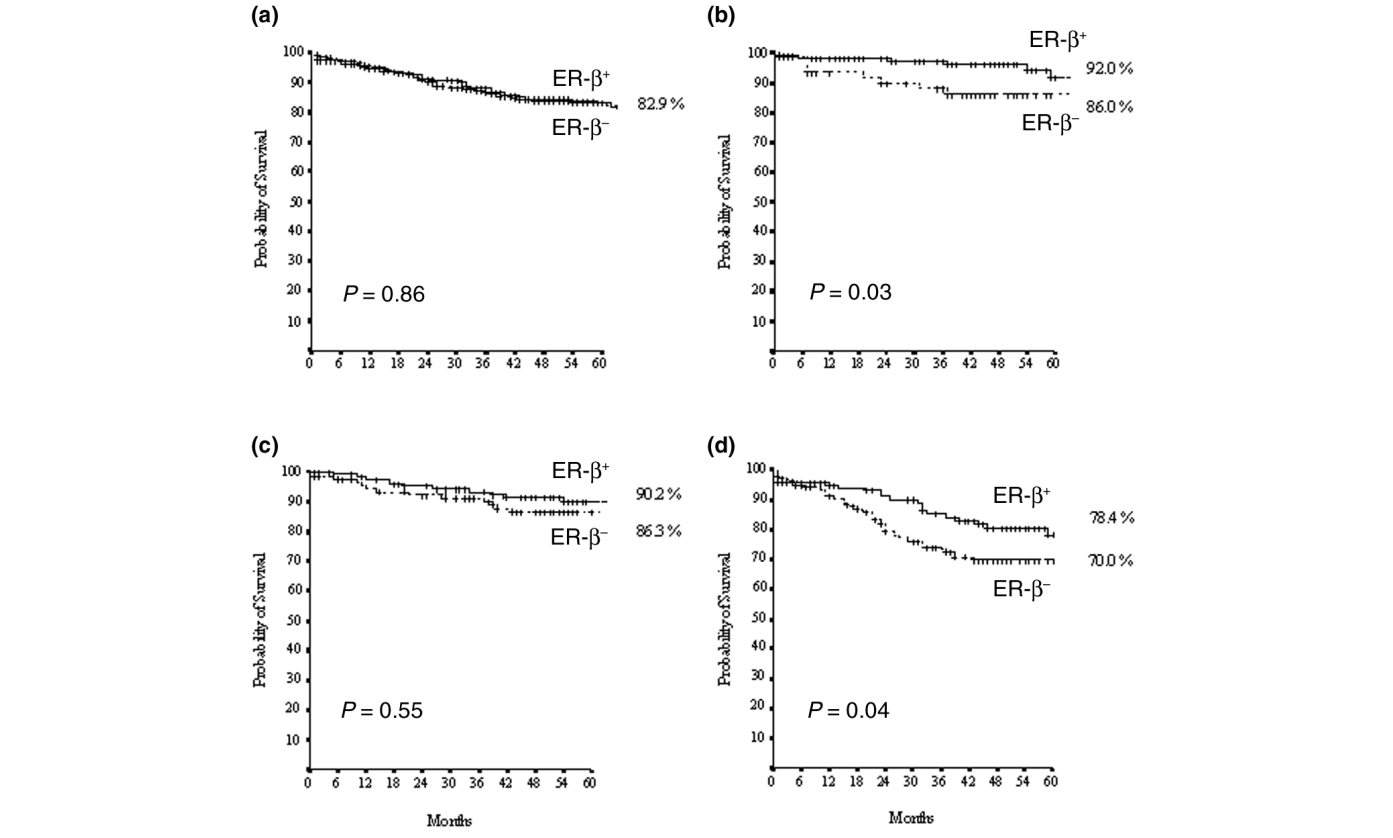
**Kaplan-Meier estimates of disease-free survival for estrogen receptor-beta (ER-β) status**. Kaplan-Meier estimates of disease-free survival for estrogen receptor-beta (ER-β) status in the whole patient group **(a)**, in the 210 node-negative patients treated exclusively with hormonal therapy **(b)**, in the 240 node-negative patients subjected to adjuvant chemotherapy followed or not followed by hormonal therapy **(c)**, and in the 246 node-positive patients subjected to adjuvant chemotherapy followed or not followed by hormonal therapy **(d)**. *P *values were calculated using the log-rank test.

**Figure 6:**
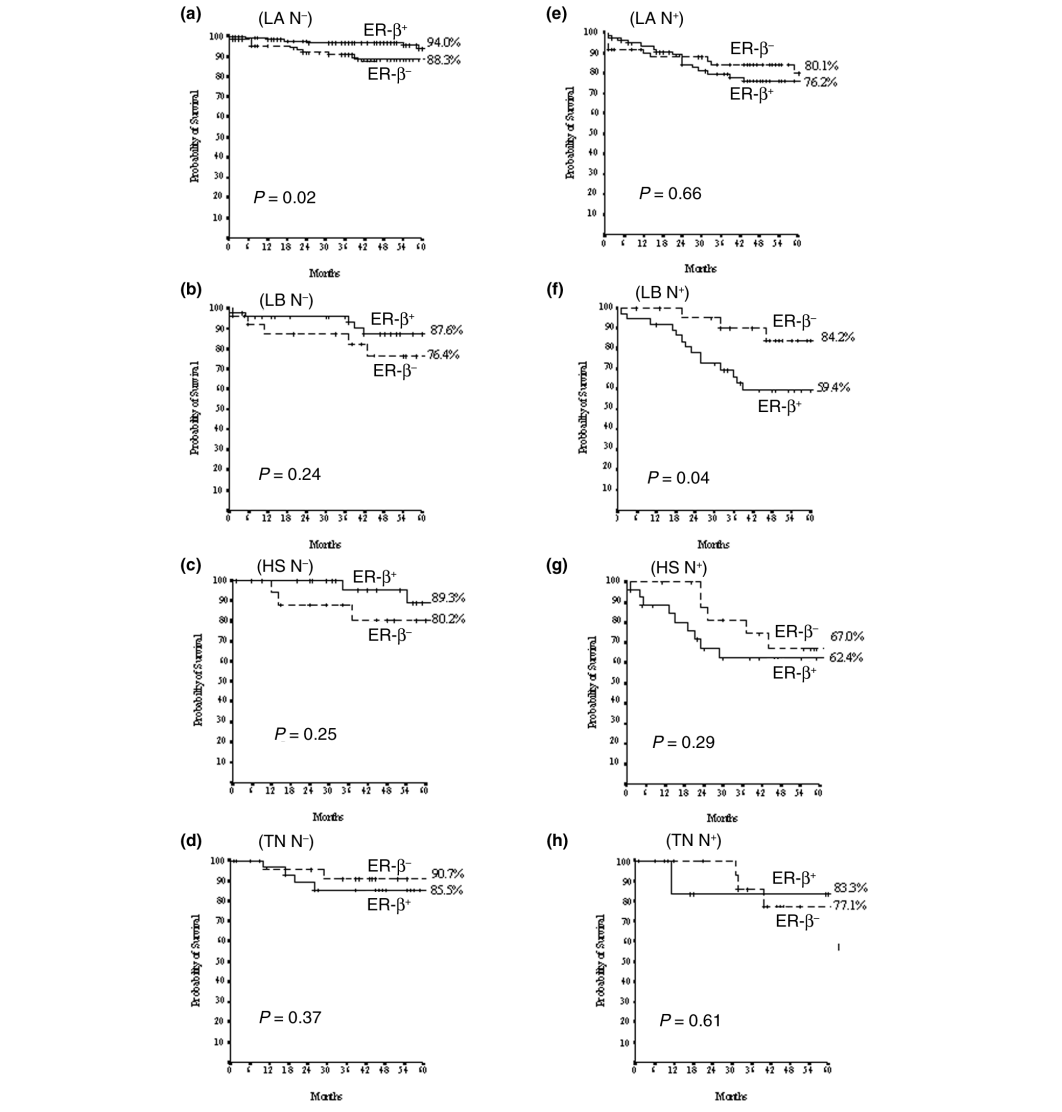
**Kaplan-Meier estimates of disease-free survival for estrogen receptor-beta (ER-β) status within the molecular subtypes**. Kaplan-Meier estimates of disease-free survival for estrogen receptor-beta (ER-β) status within the molecular subtypes and according to negative (N-) or positive (N^+^) nodal status, respectively, in each subgroup: luminal A (LA) **(a, e)**, luminal B (LB) **(b, f)**, HER2 (HS) **(c, g)**, and triple-negative (TN) **(d, h)**. *P *values were calculated using the log-rank test.

We further evaluated the prognostic/predictive impact of ER-β expression by conventional statistical methods. When the entire series of 728 cases with complete follow-up data was analyzed by unadjusted Kaplan-Meier curves, ER-β expression had no discernible effect on DFS (*P *= 0.86, Figure 5a). However, when patients were stratified by nodal status and type of treatment received, ER-β expression was able to identify subgroups of patients at different risk of relapse. Within the node-negative population, high ER-β expression predicted significantly longer DFS in the 210 patients who received HT (*P *= 0.03, Figure 5b) but not in the group of 240 patients who received adjuvant CHT (*P *= 0.55, Figure 5c). Univariate analysis (Cox model) further confirmed these data, identifying ER-β+ as a significant predictor of better DFS in the 210 node-negative patients in the HT group (hazard ratio [HR] 3.033, confidence interval [CI] 1.077 to 8.539, *P *= 0.036) but not in the 240 patients in the CHT group (HR 1.265, CI 0.586 to 2.729, *P *= 0.55). Conversely, and in agreement with the results obtained by C&RT, ER-β positivity correlated with significantly poorer DFS in the 278 node-positive patients (*P *= 0.04, Figure 5d), the vast majority of whom (246/278, 89%) received adjuvant CHT (182 AC-based and 64 non-AC-based CHT following or not by endocrine treatment). In the group of 278 N+ patients, the impact of ER-β+ on adverse clinical outcome seems to be of prognostic and not of predictive value as evidenced by Cox model (246 patients CHT-treated HR 1.58, CI 0.932 to 2.707, *P *= 0.089; 32 patients HT-treated HR 2.011, CI 0.402 to 10.057, *P *= 0.39). When the impact of ER-β expression on DFS was examined in the context of different BC molecular subtypes, it proved to be of further prognostic value in LA and LB patients, depending on their nodal status. Indeed, ER-β positivity identified patients at significantly lower probability of relapse in node-negative (*P *= 0.02, Figure 6a) but not in node-positive (*P *= 0.66, Figure 6e) LA cases. Conversely, ER-β positivity identified patients at significantly higher probability of relapse in node-positive (*P *= 0.04, Figure 6f) but not in node-negative (*P *= 0.24, Figure 6b) LB cases. No significant effect of ER-β expression on DFS was observed in HS or TN, regardless of nodal status (Figure 6c,d,g,h).

### Multivariate analyses

As summarized in Table 2, in the entire series of 728 patients with complete follow-up data, multivariate analysis revealed that tumor size (HR 2.51, CI 1.40 to 4.50, *P *= 0.002), nodal status (HR 2.44, CI 1.62 to 3.66, *P *< 0.0001), and Ki67 proliferation index (HR 1.59, CI 1.08 to 2.34, *P *= 0.02) were independent prognostic variables influencing DFS. However, in the node-positive subgroup, ER-β positivity (HR 1.55, CI 1.93 to 2.57, *P *= 0.09) emerged as an adverse prognostic factor for DFS along with tumor size (HR 2.51, CI 1.40 to 4.50, *P *= 0.002). An opposite effect was observed in the node-negative subgroup, in which ER-β negativity (HR 1.76, CI 0.95 to 3.24, *P *= 0.07) and elevated Ki67 proliferation index (HR 2.16, CI 1.15 to 4.04, *P *= 0.02) were associated with a poorer disease outcome.

**Table 2:** Multivariate analyses of negative prognostic factors for disease-free survival

	Disease-free survival	
	
Factors	HR (95% CI)	*P *value
All patients (728)		
pT stage (3–4 versus 1–2)	2.51 (1.40–4.50)	0.002
pN stage (positive versus negative)	2.44 (1.62–3.66)	<0.0001
Ki67 (positive versus negative)	1.59 (1.08–2.34)	0.02
Node-positive patients (278)		
pT stage (3–4 versus 1–2)	2.51 (1.40–4.50)	0.002
ER-β (positive versus negative)	1.55 (1.93–2.57)	0.09
Node-negative patients (450)		
ER-β (negative versus positive)	1.76 (0.95–3.24)	0.07
Ki67 (positive versus negative)	2.16 (1.15–4.04)	0.02

Cox regression analysis using a forward stepwise procedure (enter limit = 0.10, remove limit = 0.15). CI, confidence interval; ER-β, estrogen receptor-beta; HR, hazard ratio; pN, pathological node status; pT, pathological tumor size.

## Discussion

To the best of our knowledge, this is the first study in which the relationship between ER-β expression, established biopathological factors, and patient outcome was investigated in an observational prospective study including a large series of invasive BC, consecutively accrued over a relatively limited and recent period of time (2001 to 2005). While ER-α and PgR expression was significantly associated with HER2, Ki67, p53, Bcl2, T, N, and G, as one would expect in a representative well-balanced cohort of unselected patients with early BC, we observed no significant association between ER-β expression and the classical biopathological parameters. Our findings, while in agreement with other recently published studies [3,18], differ from others that have found that ER-β is coexpressed with ER-α and PgR and is associated with nodal status, grade, proliferation rate [17,19], and HER2 overexpression [20]. Inconsistencies among different studies are possibly due to different techniques in determining ER-β expression and lack of validated reagents for IHC. Jarvinen and colleagues [17] and Umekita and colleagues [20] used the polyclonal antibody PAI-313 and the MoAb EMR02, respectively, whereas Omoto and colleagues [19] did not specify the reagents used for their IHC analysis. In our study, we used two well-characterized anti-ER-β MoAbs, PPG5/10 (ER-β1) and 14C8 (total ER-β), which have previously been shown to be the best performing antibodies for IHC staining [21] with superimposable results. Moreover, further discrepancies could also be related to the selection of different cohorts of patients. In fact most authors included in their study only retrospective series of BC patients dating back to the early 90s, whereas our series is prospective and includes patients treated between 2001 and 2005.

We took this kind of analysis one step further and also examined the distribution of ER-β among different, molecularly distinct, BC subtypes. Gene expression profiling [8] has, in fact, led to the identification of subtypes of invasive BC with different outcomes, namely LA, LB, TN, and HS. Such classification has since been translated into routine clinical practice by combining a limited set of markers (ER, PgR, HER2, and basal cytokeratins) that can be assessed by IHC [9]. We stratified our 936 BC patients according to these molecular subtypes and found that ER-β evenly distributes across the four subtypes, as recently reported by other authors [22]. Such results were confirmed by MCA [23,24], an alternative method for analyzing multiple categorical variables by graphically visualizing their interrelationships [25], which showed that ER-β expression presents a limited dispersion around the origin, regardless of the method used to classify all of the variables, that is, by clustering them into discrete subgroups (Figure 3a) or considering them individually (Figure 3b). These findings strongly support the lack of correlation between ER-β expression and the other biopathological parameters considered, further validating the hypothesis that ER-β has functions that are distinct from those of ER-α [26].

The lack of association between ER-β and other classical prognostic factors makes it an even more attractive candidate as a prognostic/predictive biomarker. The impact of ER-β expression on disease outcome (in terms of DFS) was therefore studied in a subset of 728 patients with a median follow-up of 50 months. Using a nonparametric statistical procedure, C&RT analysis, we were able to identify ER-β as a discriminating factor in two very interesting subgroups of patients: (a) node-positive patients, in whom ER-β^+ ^appears to convey a higher risk of relapse, particularly when coupled with PgR negativity, and (b) node-negative patients, in whom ER-β^+ ^appears to predict a favorable response to endocrine therapy. These results were substantially validated by conventional statistical procedures, such as Kaplan-Meier analysis of DFS curves and univariate and multivariate Cox regression analysis, both of which seem to indicate a divergent role of ER-β expression as a positive predictive factor in node-negative patients subjected to HT as well as a negative prognosticator in node-positive patients which does not predict the response to any therapeutic regimen. Though based on a limited number of DFS events, the finding of a positive influence of ER-β expression on the outcome of node-negative BC patients treated exclusively with HT is supported by several other reports in which the predictive value of ER-β, detected by mRNA or IHC staining, was investigated in BC patients undergoing endocrine therapy [3,27,28]. In these studies, positive ER-β protein staining was invariably almost associated with a favorable response to antiestrogen treatment, consistent with its antiproliferative and anti-invasive properties observed in ER-β-expressing cell lines [29]. Conversely, to the best of our knowledge, this is the first study in which ER-β expression is unexpectedly found to be significantly associated with an unfavorable prognosis in node-positive in an observational prospective series of BC patients. This is in agreement with data reported in prostate cancer providing evidence that ER-β^+ ^tumors had a higher rate of relapse and a small but significant decrease in relapse-free survival compared with those in which ER-β expression had been lost [30]. One likely explanation for our findings in BC is that all of the previous studies that have measured ER-β in BC have focused on response to tamoxifen therapy in either adjuvant or metastatic settings [26,31], while our subset of node-positive BC patients mostly received adjuvant CHT (with or without ACs). It is interesting to note that the divergent role of ER-β expression is maintained even when established pathological factors are clustered together into distinct molecular subgroups in the context of a widely used clinical translation of gene expression profiling studies (LA, LB, TN, and HS). Indeed, depending on nodal status, ER-β expression might usefully complement the prognostic assessment of patients in those subgroups (LA and LB) where further risk stratification by gene expression analysis is needed to accurately predict prognosis [2,20]. In this context, ER-β positivity might signal responsiveness to hormonal treatment in node-negative LA patients, on one hand, and a more aggressive clinical course, requiring *ad hoc *tailored therapeutic interventions, in node-positive LB patients, on the other, thereby possibly contributing to the implementation of individualized therapeutic strategies.

## Conclusion

These data support the continued prospective investigation of ER-β expression in BC patients, adding novel insights into the complex mechanisms underlying the endocrine pathway. Future investigations will need to assess the role of ER-β, taking into account an everchanging scenario in which both ER biology and endocrine treatment paradigms for BC are rapidly evolving as highlighted by the discovery of multiple ER-β variants [32] and by the increasing inclusion of aromatase inhibitors, either upfront or in switching/sequencing strategies, in the adjuvant treatment plan of patients with early BC [33].

## Abbreviations

AC: anthracycline; BC: breast cancer; C&RT: classification and regression tree; CHT: chemotherapy; CI: confidence interval; DFS: disease-free survival; EGFR: epidermal growth factor receptor; ER-α: estrogen receptor-alpha; ER-β: estrogen receptor-beta; G: histological grade; HR: hazard ratio; HS: HER2; HT: hormonal therapy; IHC: immunohistochemistry; LA: luminal A; LB: luminal B; MCA: multiple correspondence analysis; MoAb: monoclonal antibody; N: lymph node status; PgR: progesterone receptor; T: tumor size; TN: triple-negative.

## Competing interests

The authors declare that they have no competing interests.

## Authors' contributions

FN and M Milella were responsible for the study design; produced, acquired, analyzed, and interpreted the data; and drafted the manuscript. FN and M Milella contributed equally to this work. EM and SB provided assistance in data acquirement and interpretation and in manuscript drafting. IS was responsible for the database set-up and for the statistical analyses. LP and RP-D revised all the slides submitted to immunohistochemical staining and confirmed the histological diagnosis. IV, CN, AF, and ADB provided clinical data of the patients, including treatment schedule and follow-up. PGN critically revised the manuscript for important intellectual content. M Mottolese was responsible for the study concept and design and for the interpretation of results, helped in data discussion, critically revised the manuscript for important intellectual content, and obtained funding for the study. All authors read and approved the final manuscript.

## Supplementary Material

Additional file 1 Classification and Regression Tree (C&RT) analysis is applied to generate the ER-beta cut off percentage. Diagram shows diagnostic algorithm, generated by Answer-Tree 3.1 software, Statistical Package for the Social Sciences. We introduced into the model three variables (nodal status, ER-beta expression, relapses). The model indicated that in node negative patients the highest percentage of relapses occurred when ER-beta was positive in less than 17.5% of neoplastic cells whereas in node positive patients the highest percentage of relapses occurred when ER-beta was positive in more than 12.5% of neoplastic cells. We chose as cut off 20% being the percentage of ER-beta positivity which includes both values. **Abbreviations**: N-, node negative; N+, node positive; No = absence of recurrences; Yes = presence of recurrences. Click here for file

Additional file 2 Distribution of the 109 recurrences occurred in the 728 breast cancer patients analyzed for DFS. The table summarizes the type and the site of recurrences including local, distant metastases and contralateral cancers. Click here for file
