# Supplementary figures and images for: A divergent role for estrogen receptor-beta in node-positive and node-negative breast cancer classified according to molecular subtypes: an observational prospective study
Source: Breast Cancer Res. 2008 Sep 4;10(5):R74. doi: 10.1186/bcr2139 (PMC2614505; doi:10.1186/bcr2139)

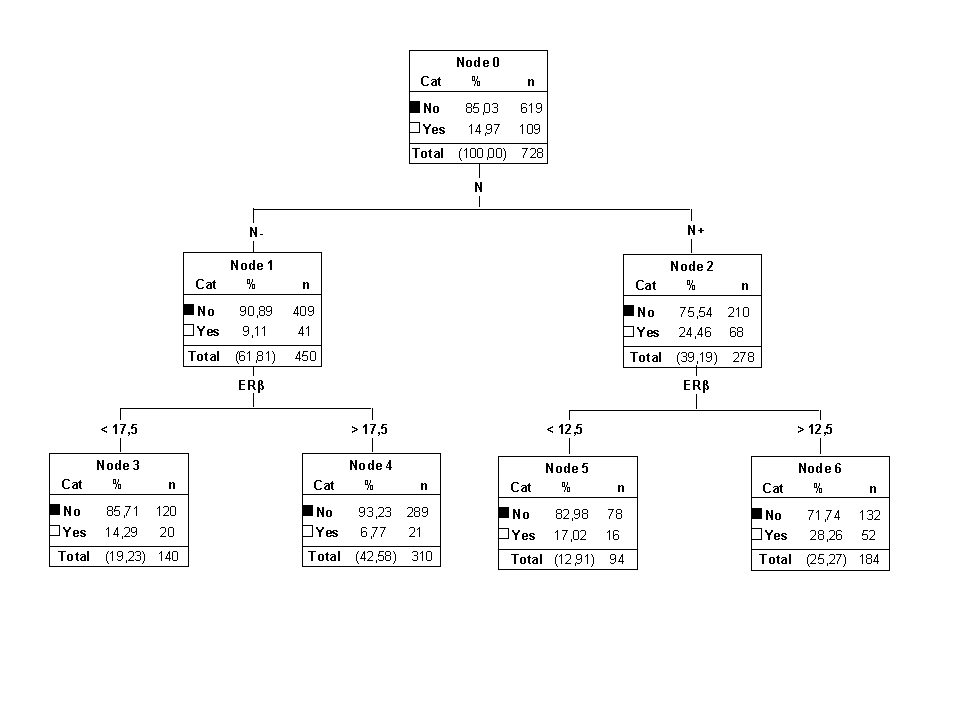

Supplement: Additional file 1 — Classification and Regression Tree (C&RT) analysis is applied to generate the ER-beta cut off percentage. Diagram shows diagnostic algorithm, generated by Answer-Tree 3.1 software, Statistical Package for the Social Sciences. We introduced into the model three variables (nodal status, ER-beta expression, relapses). The model indicated that in node negative patients the highest percentage of relapses occurred when ER-beta was positive in less than 17.5% of neoplastic cells whereas in node positive patients the highest percentage of relapses occurred when ER-beta was positive in more than 12.5% of neoplastic cells. We chose as cut off 20% being the percentage of ER-beta positivity which includes both values. Abbreviations: N-, node negative; N+, node positive; No = absence of recurrences; Yes = presence of recurrences. [file bcr2139-S1.bmp]
