# Supplementary material for: A divergent role for estrogen receptor-beta in node-positive and node-negative breast cancer classified according to molecular subtypes: an observational prospective study
Source: Breast Cancer Res. 2008 Sep 4;10(5):R74. doi: 10.1186/bcr2139 (PMC2614505; doi:10.1186/bcr2139)
Supplement: Additional file 2 — Distribution of the 109 recurrences occurred in the 728 breast cancer patients analyzed for DFS. The table summarizes the type and the site of recurrences including local, distant metastases and contralateral cancers. [file bcr2139-S2.rtf]

Additional File 2

Distribution of the 109 recurrences occurred in the 728 Breast Cancer patients analyzed
for DFS

RECURRENCES	N° of cases	%	
Local 	11	10.1	
Visceral 	57	52.4	
Bone 	20	18.4	
Contralateral cancer	6	5.4	
Other*	15	13.7	
Total	109	100	
	     
	     * Including lymph node, central nervous system and multiple site metastases
